# The essential role of jasmonate signaling in *Solanum habrochaites* rootstock-mediated cold tolerance in tomato grafts

**DOI:** 10.1093/hr/uhac227

**Published:** 2022-10-11

**Authors:** Lihui Wang, Bo Wu, Guoyu Chen, Hui Chen, Yuquan Peng, Hamza Sohail, Shouyu Geng, Guangbao Luo, Dandi Xu, Bo Ouyang, Zhilong Bie

**Affiliations:** Key Laboratory of Horticultural Plant Biology, Ministry of Education, College of Horticulture and Forestry Sciences, Huazhong Agricultural University, Wuhan, 430070, P.R. China; Key Laboratory of Horticultural Plant Biology, Ministry of Education, College of Horticulture and Forestry Sciences, Huazhong Agricultural University, Wuhan, 430070, P.R. China; Key Laboratory of Horticultural Plant Biology, Ministry of Education, College of Horticulture and Forestry Sciences, Huazhong Agricultural University, Wuhan, 430070, P.R. China; Key Laboratory of Horticultural Plant Biology, Ministry of Education, College of Horticulture and Forestry Sciences, Huazhong Agricultural University, Wuhan, 430070, P.R. China; Key Laboratory of Horticultural Plant Biology, Ministry of Education, College of Horticulture and Forestry Sciences, Huazhong Agricultural University, Wuhan, 430070, P.R. China; Key Laboratory of Horticultural Plant Biology, Ministry of Education, College of Horticulture and Forestry Sciences, Huazhong Agricultural University, Wuhan, 430070, P.R. China; Key Laboratory of Horticultural Plant Biology, Ministry of Education, College of Horticulture and Forestry Sciences, Huazhong Agricultural University, Wuhan, 430070, P.R. China; Key Laboratory of Horticultural Plant Biology, Ministry of Education, College of Horticulture and Forestry Sciences, Huazhong Agricultural University, Wuhan, 430070, P.R. China; Key Laboratory of Horticultural Plant Biology, Ministry of Education, College of Horticulture and Forestry Sciences, Huazhong Agricultural University, Wuhan, 430070, P.R. China; Key Laboratory of Horticultural Plant Biology, Ministry of Education, College of Horticulture and Forestry Sciences, Huazhong Agricultural University, Wuhan, 430070, P.R. China; Key Laboratory of Horticultural Plant Biology, Ministry of Education, College of Horticulture and Forestry Sciences, Huazhong Agricultural University, Wuhan, 430070, P.R. China

## Abstract

Tomato (*Solanum lycopersicum*) is among the most important vegetables across the world, but cold stress usually affects its yield and quality. The wild tomato species *Solanum habrochaites* is commonly utilized as rootstock for enhancing resistance against abiotic stresses in cultivated tomato, especially cold resistance. However, the underlying molecular mechanism remains unclear. In this research, we confirmed that *S. habrochaites* rootstock can improve the cold tolerance of cultivated tomato scions, as revealed by growth, physiological, and biochemical indicators. Furthermore, transcriptome profiling indicated significant differences in the scion of homo- and heterografted seedlings, including substantial changes in jasmonic acid (JA) biosynthesis and signaling, which were validated by RT–qPCR analysis. *S. habrochaites* plants had a high basal level of jasmonate, and cold stress caused a greater amount of active JA-isoleucine in *S. habrochaites* heterografts. Moreover, exogenous JA enhanced while JA inhibitor decreased the cold tolerance of tomato grafts. The JA biosynthesis-defective mutant *spr8* also showed increased sensitivity to cold stress. All of these results demonstrated the significance of JA in the cold tolerance of grafted tomato seedlings with *S. habrochaites* rootstock, suggesting a future direction for the characterization of the natural variation involved in *S. habrochaites* rootstock-mediated cold tolerance.

## Introduction

Being sessile in nature, plants are vulnerable to various environmental stresses. Low temperature, often known as cold stress, is a significant barrier to plant growth and development, causing leaf wilting, yellowing, and even necrosis [1]. Cultivated tomato (*Solanum lycopersicum*) is native to the tropics and subtropics and is susceptible to cold stress. In contrast, the wild tomato *Solanum habrochaites* can withstand low temperature and even frost [2]. Compared with cultivated tomato, *S. habrochaites* has thick hairs on the leaf surface, which may contribute to cold tolerance [3]. In addition, the cold tolerance of this wild species may be regulated by various physiological and biochemical processes, such as membrane structure, membrane lipid metabolism, and other metabolic regulations [3, 4]. Since there is a strong reproductive barrier between *S. habrochaites* and *S. lycopersicum*, an alternative approach to utilizing the resistance of *S. habrochaites* is grafting.

Grafting is an agronomic technique intensively used in the horticultural industry to boost plant resistance toward biotic and abiotic stresses [5]. Grafting is extensively utilized in tomato to boost yield or quality, while also increasing resistance to a variety of stressors, including cold stress and root-knot disease [6, 7]. The improvement of cold tolerance by grafting onto cold-tolerant rootstock can be attributed to several physiological mechanisms: (i) avoidance of damage by closing stomata more quickly than sensitive genotypes when temperature decreases [8]; (ii) improvement of scion tolerance by increasing osmoprotectants (free proline, betaine, and soluble sugar) in the cytosol [9]; (iii) elimination of oxygen radicals by increasing the contents of detoxifying substances (glutathione) or antioxidant enzymes (peroxidase and ascorbate peroxidase) [10]; (iv) modulation of cold tolerance by affecting photosynthesis, and carbon and nitrogen metabolism [11]; and (v) maintenance of stomatal conductance and nitrogen content, thus achieving better plant establishment and growth performance at suboptimal temperatures [12]. However, investigations on the molecular mechanism of enhancing tomato cold tolerance through grafting are rare. Plants’ abiotic stress tolerance is greatly influenced by root–shoot communication [13]. Rootstock can improve scion tolerance through long-distance communication signals, such as Ca^2+^ signals, reactive oxygen species (ROS), plant hormones, water and nutrients, RNA, and peptides [14, 15]. However, it remains largely unknown how these signaling molecules affect plant stress tolerance in grafted plants. Overexpression of *SlNCED1* in tomato rootstock increases the abscisic acid (ABA) concentration in scions and alleviates salinity stress via root–shoot communication during salinity stress [13]. Cotton roots trigger a significant quantity of jasmonic acid (JA) synthesis in leaves, and subsequently transport it to the roots in response to osmotic stress, which upregulates plasma membrane intrinsic protein (PIP) proteins in roots and improves root water uptake [16]. Therefore, JA can function as a signal in graft-mediated stress tolerance.

The biosynthesis and signaling of JA in plants have received extensive attention, and JA-related compounds are collectively termed jasmonates. The substrate for JA production is the unsaturated fatty acid α-linolenic acid (18:3), which is liberated from the sn1 position of galactolipids on plastid membranes by the action of PLA1 (phospholipase A1). The next step involves the successive operations of LOX (13-lipoxygenase), AOS (allene oxide synthase), and AOC (allene oxide cyclase) to transform the substrate into OPDA [(9S, 13S)-12-oxo-phytodienoic acid]. OPDA is then transported from the plastid to the peroxisome, where it undergoes three oxidation processes by the enzymes ACX (acyl-CoA oxidase), MFP (multifunctional protein), and KAT (l-3-ketoacyl CoA thiolase) to become (+)-7-iso-JA. Finally, under the influence of the JA-Ile synthesizing enzyme (JAR1), (+)-7-iso-JA is transported to the cytoplasm, where it is conjugated with isoleucine (Ile) to create (+)-7-iso-JA-Ile. The most bioactive JA molecule is JA-Ile, which is taken into the nucleus by the ABC transporter JAT1 and contributes to the later steps of the JA signaling cascade [17]. Plants have very low JA-Ile concentration under normal conditions, and the jasmonate ZIM-domain (JAZ) protein inhibits the expression of JA-responsive genes by interacting with several transcription factors (among them, MYC2 is the most studied). However, biotic/abiotic stresses may trigger JA-synthesizing gene expression and raise JA contents [18, 19]. The accumulated JA conjugates with isoleucine to form JA-Ile, which can promote the ubiquitination and degradation of JAZ by binding to the receptor coronatine insensitive 1 (COI1), thus activating the JA signaling pathway [20]. Some enzymes (such as LOX, AOS, JAR1, and MYC2) are particularly important in JA synthesis and signaling. It has been experimentally demonstrated that the *spr8* mutant caused by a point mutation in the catalytic domain of *LoxD* exhibits a series of JA-dependent immune defects, such as abnormal glandular trichome development, inability to express trauma-responsive genes, and severe reduction of resistance to *Botrytis cinerea* and *Helicoverpa armigera* [21]. Similarly, the activities of disease resistance and antioxidant enzymes are markedly reduced by *SlMYC2* deletion, which aggravates disease symptoms in tomato [22].

Recent studies in *Arabidopsis thaliana* and other plants have shown evidence that JA affects how the plant reacts to cold stress [19]. Cao *et al*. [23] found that during the storage of loquat fruits treated with methyl jasmonate (MeJA) , the levels of antioxidative enzymes, including SOD (superoxide dismutase), APX (ascorbate peroxidase), and CAT (catalase), increased, but the activity of lipoxygenase decreased, which reduces chilling injury of the fruits. In banana fruits, *MaMYC2* and *MaICE1* function synergistically in MeJA-induced cold tolerance [24]. The JA signaling system greatly enhances cold resistance in apple (*Malus hupehensis*) by the JAZ–BBX37–ICE1–CBF pathway [25]. Furthermore, JA has been demonstrated to improve cold adaptation by enhancing the production of osmotic substances, such as glycine betaine in *Poncirus trifoliata* [26] and polyamine in tomato [27].

In the tomato industry, *S. habrochaites* accession LA1777-derived rootstocks are used to improve cold tolerance. However, the underlying molecular mechanism remains to be elucidated. It has been accepted that LA1777 has stronger cold tolerance than cultivated tomato [4]. LA1777 rootstock can alleviate the damage to the growth and development of cultivated tomato under sub-low temperature (15°C) conditions [2, 28]. It remains to be determined whether the grafts can survive in harsher conditions (4°C), and the physiological and molecular mechanisms of LA1777 as a rootstock for improving tomato cold tolerance require additional investigation. In this study, we used *S. habrochaites* LA1777 as rootstock and cultivated tomato as scion to investigate the mechanism of tomato grafts under cold stress. It was discovered that enhanced JA synthesis in scions played a crucial role in grafted tomato cold tolerance when *S. habrochaites* was used as rootstock.

## Results

### *Solanum habrochaites* rootstock improves the cold tolerance of cultivated tomato scion

**Figure 1:**
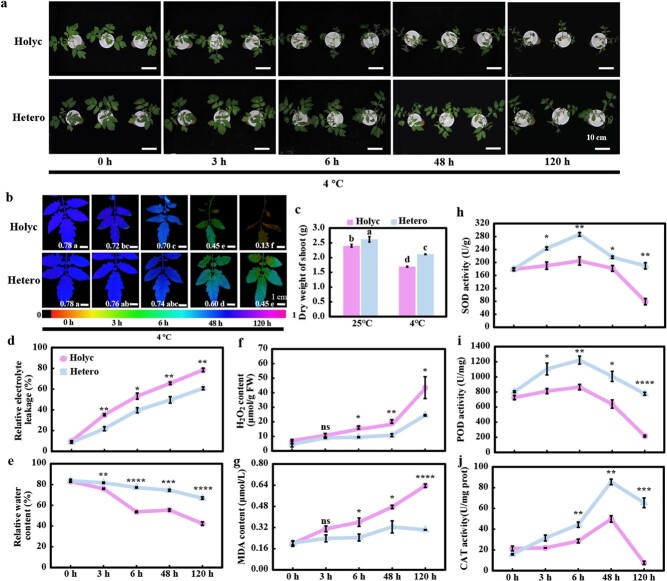
*S. habrochaites* rootstock alleviates damage of cultivated tomato scion under cold stress through enhancing antioxidant enzymes. **a** Phenotype of grafted seedlings under chilling stress. **b** Images of the maximum photochemical efficiency of PSII (*F*_v_*/F*_m_). The false-color code depicted at the bottom of the image ranges from 0 (black) to 1 (purple). Means denoted with different letters differ significantly at *P* < .05. **c** Dry weight of the grafted plant scion. **d** Relative electrolyte leakage. **e** Relative water content. **f** H_2_O_2_ content. **g** MDA content. **h**–**j** SOD (**h**), POD (**i**), and CAT (**j**) activities. *S. lycopersicum* seedlings grafted onto rootstock of *S. lycopersicum* (Holyc) or *S. habrochaites* (Hetero) were treated with cold at 4°C. The data in **a**, **b**, and **d**–**j** were obtained 0, 3, 6, 48 and 120 hours after cold exposure, respectively; the data in **c** were obtained 120 hours after cold exposure. Data are means of three biological replicates (± standard deviation) with three technical replicates each. Statistical significance levels (Student’s *t*-test) in **d**–**j**: ns, not significant; ^*^P ≤ .05; ^**^P ≤ .01; ^***^P ≤ .001, ^****^P ≤ .0001.

To assess the potential contribution of *S. habrochaites* LA1777 rootstock to the cold tolerance of scions, we first constructed four grafting combinations of *S. lycopersicum* and *S. habrochaites* by homo- and heterografting. It was found that under 12 hours of cold stress plants with *S. habrochaites* rootstock outperformed those with cultivated tomato rootstock (Supplementary Data Fig. S1a), which was supported by various stress-related indicators, including relative electrical conductivity (REL) (Supplementary Data Fig. S1b), malondialdehyde (MDA) content (Supplementary Data Fig. S1c) and relative water content (RWC) (Supplementary Data Fig. S1d). Moreover, we evaluated the cold tolerance of homografted *S. lycopersicum* LA4024 (Holyc) and heterografted plants (LA4024/LA1777, abbreviated as Hetero) by comparing plant performance and the stress-related indicators, including the maximum photochemical efficiency of PSII (*F*_v_*/F*_m_) values, dry weight, RWC, and REL (Fig. 1). Under normal conditions, no significant difference was observed in *F*_v_*/F*_m_, RWC, and REL between homo- and heterografts, although the dry weight of Hetero seedlings was slightly higher than that of Holyc seedlings (time point 0 in Fig. 1). When exposed to 4°C, both Holyc and Hetero seedlings showed a wilting phenotype, a decline in *F*_v_*/F*_m_ and RWC, and an increase in REL (Fig. 1a–e). However, the cold-induced wilting, *F*_v_*/F*_m_ decline, and REL rise in the scion leaves were significantly reduced by LA1777 rootstock. For example, REL was significantly lower while RWC was significantly higher at all the time points in the scion leaves of Hetero than in those of Holyc (Fig. 1d and e).

Several stress-related biochemical indicators were further compared, including ROS accumulation, MDA metabolism, and the activity of antioxidant enzymes POD, (Peroxidase) SOD, and CAT. Under cold treatment, overproduction of ROS and higher lipid peroxidation (as indicated by MDA) were detected in the scion of Holyc compared with that of Hetero (Fig. 1f and g). Under normal conditions, the activities of POD, SOD, and CAT showed no significant difference between Holyc and Hetero. However, when the seedlings were exposed to 4°C the activities of these enzymes first increased and then decreased, but they were much higher in the scion of Hetero than in that of Holyc. Especially at the late stage of treatment (120 hours), when the antioxidant enzyme activity of Holyc seedlings was barely detectable, it was retained at a relatively higher level in Hetero seedlings (Fig. 1h–j).

### Transcriptome differences in tolerant and sensitive scions under cold stress

**Figure 2:**
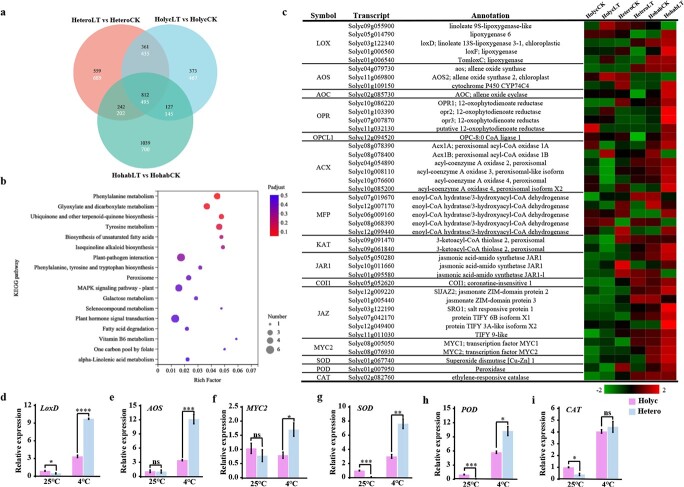
Differences in gene expression between tolerant and sensitive scions under cold stress. **a** Venn diagram of DEGs in scions in the comparisons between HolycLT and HolycCK, HeteroLT and HeteroCK, and HohabLT and HohabCK. The black and white numbers represent numbers of upregulated and downregulated genes, respectively. **b** Enriched KEGG pathways based on cold-responsive upregulated genes exclusively identified in both Hohab and Hetero scions. Colored panels illustrate the significance level of enrichment. **c** Heat map of JA biosynthesis and signaling genes responsive to JA in Holyc, Hetero, and Hohab scions after 3 hours of 4°C treatment. Colored panels display the log_2_ value of fold change. **d**–**i** Expression analysis of related genes (**d***LOXD*; **e***AOS*; **f***MYC2*; **g***SOD*; **h***POD*; **i***CAT*.) in cold-treated tomato scions by RT–qPCR. *S. lycopersicum* homografting (Holyc), *S. lycopersicum* seedlings grafted onto rootstock of *S. habrochaites* (Hetero), and *S. habrochaites* homografts (Hohab) were treated at 4°C (LT), with 25°C treatment as control. Statistical significance levels (Student’s *t*-test): ns, not significant; ^*^P ≤ .05; ^**^P ≤ .01; ^***^P ≤ .001; ^****^P ≤ .0001.

For investigation of the difference in expression profiles of cold-tolerant (Hohab and Hetero) and sensitive (Holyc) grafts in response to cold stress, we performed a comparative transcriptomic analysis using scion samples from 3 hours of cold treatment. A total of 3762 (2220 up- and 1542 downregulated), 3795 (1974 up- and 1821 downregulated), and 3215 (1673 up- and 1542 downregulated) cold-responsive genes (CRGs) [log_2_|(cold stress/control)| ≥ 1, *q*-value <.05] were identified in the scion of Hohab, Hetero, and Holyc, respectively. In comparison with Holyc, Hohab and Hetero displayed a larger number of CRGs. A total of 1307 CRGs (812 up- and 495 downregulated) were shared by all three grafting combinations, whereas 444 CRGs (242 up- and 202 downregulated) were exclusively shared by Hohab and Hetero. These 444 CRGs potentially suggest differential expression under cold stress using *S. lycopersicum* LA4024 or *S. habrochaites* LA1777 rootstock (Fig. 2a). Gene Ontology (GO) enrichment of the 242 upregulated CRGs showed that several biological processes were enriched, including cellular response to extracellular stimulus, cellular response to external stimulus, and cell communication (Supplementary Data Fig. S2a), while enriched GO terms for the 202 downregulated CRGs included protein phosphorylation and protein modification process (Supplementary Data Fig. S2b). These CRGs were considerably abundant in the biosynthesis of unsaturated fatty acids, α-linolenic acid metabolism, and plant hormone signal transduction, according to Kyoto Encyclopedia of Genes and Genomes (KEGG) pathway analysis (Fig. 2b), highlighting a connection with JA biosynthesis and signal transduction. Therefore, we conducted a heat map analysis for the genes involved in JA biosynthesis and signal transduction. It was found that there were more genes upregulated and the fold change was generally higher in Hohab and Hetero scion than in Holyc scion under cold stress. For instance, *LoxD* (Solyc03g122340) was upregulated 12.4-fold in Hohab and 7.3-fold in Hetero under cold stress, but only 3.6-fold in Holyc (Fig. 2c).

As JA has been associated with cold tolerance, we studied how it affects the cold resistance of grafted tomato seedlings. We first made an overlap between the different sets of CRGs mentioned above and the MYC2 targets. MYC2 targets were obtained by analyzing publicly accessible RNA-seq data (PRJCA000395, National Genomics Data Center), where chromatin immunoprecipitation sequencing (ChIP-seq) coupled with RNA sequencing identified 665 MYC2-targeted JA-responsive (MTJA) genes [29]. The results showed that there were 166, 123, and 100 overlapping genes with the 655 MTJA genes in the upregulated CRGs of Hohab, Hetero and Holyc, respectively (Supplementary Data Fig. S2c). GO enrichment of the overlapped genes for all three grafting combinations (Holyc, Hetero, Hohab) showed that the enriched molecular function terms included DNA-binding transcription factor activity and lyase activity, while the enriched terms only for Hetero and Hohab included catalytic activity, metal ion binding, etc. (Supplementary Data Fig. S2d). We found that three antioxidant defense genes responsive to JA were enriched for Hetero and Hohab but not Holyc: Solyc01g067740 (*SOD*), Solyc01g007950 (*POD*), and Solyc02g082760 (*CAT*). We included these JA-regulated genes in the heat map in order to better understand their function in the tomato’s cold response (Fig. 2c). It was shown that after 3 hours of cold stress, *SOD* and *CAT* were significantly increased to variable degrees. Additionally, RT–qPCR was used to further confirm the expression pattern of several JA biosynthesis andsignaling pathway genes (*LoxD*, *AOS*, and *MYC2*) (Fig. 2d–f). Consistent with the RNA-seq findings, the transcript level of JA biosynthesis genes was upregulated in all grafted seedlings after 3 hours of cold exposure, especially in Hetero. Notably, the relative expression of *LoxD* in Hetero was 9.72, 3.2-fold higher than in Holyc (Fig. 2d). We also validated antioxidative gene expression by RT–qPCR, and the results complemented the transcriptome data (Fig. 2g–i). Since the major differences in gene expression in tomato scions were due to differences in rootstocks, our transcriptome results revealed that JA is likely to play a key role in this difference by regulating antioxidant enzyme metabolism.

### Different rootstocks induced jasmonic acid accumulation differently in scions of cultivated tomato plants under cold stress

**Figure 3:**
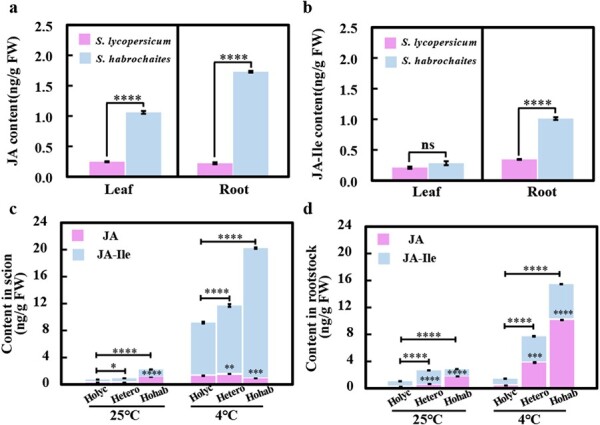
High basal level of JA in *S. habrochaite* rootstock may contribute to scion cold tolerance. **a**, **b** Basal JA (**a**) and JA-Ile (**b**) content in *S. habrochaites* LA1777 and *S. lycopersicum* LA4024 seedlings. **c**, **d** JA and JA-Ile content in the scion (**c**) and rootstock (**d**) of homografted LA4024 (Holyc) and LA1777 (Hohab), and heterografted seedlings of LA4024/LA1777 (Hetero). Five-leaf tomato plants were subjected to cold stress at 4°C for 3 hours. After treatment, leaves were harvested for the determination of JA and JA-Ile content. Data are means of three replicates (± standard deviation). Statistical significance levels (Student’s *t*-test): ns, not significant; ^*^P ≤ .05; ^**^P ≤ .01; ^***^P ≤ .001; ^****^P ≤ .0001.

Firstly, we determined JA and JA-Ile contents in the seedlings of *S. habrochaites* LA1777 and *S. lycopersicum* LA4024. Remarkably, LA1777 had much higher JA and JA-Ile contents in its leaves and roots than LA4024 under the normal condition. The JA content of LA1777 leaves and roots was 4.2- and 8.6-fold that of LA4024, respectively; the JA-Ile content in LA1777 roots was 1.012 ng/g fresh weight (FW), which is 2.9-fold of that in LA4024 (Fig. 3a and b). In addition, to verify the correlation between JA and the expression of antioxidative genes, we also carried out RT–qPCR on *MYC2* and the three antioxidant genes (*SOD*, *POD*, and *CAT*). The results showed that, under normal conditions, the transcription of *SOD*, *CAT*, and *MYC2* was at a slightly higher level in LA1777 than in LA4024. But under low temperature the expression of the four genes in LA1777 was significantly higher than that in LA4024 (Supplementary Data Fig. S3). These results demonstrated that the basal JA content of *S. habrochaites* LA1777 was higher than that of *S. lycopersicum* LA4024, suggesting that JA might be involved in *S. habrochaites*’ ability to withstand cold.

We also examined JA/JA-Ile accumulation in the different grafting combinations following 3 hours of cold treatment. Under normal temperature, the JA/JA-Ile contents in Holyc and Hetero scions were comparable to and lower than those in Hohab scions, respectively; in the rootstock, the content of JA and JA-Ile was the lowest in Holyc and the highest in Hohab, which was consistent with the results of self-rooted plants (Fig. 3c and d). The scions of both *S. lycopersicum* LA4024 and *S. habrochaites* LA1777 rootstocks produced more JA and had higher relative expression levels of *LoxD* and *AOS*, the key genes involved in JA biosynthesis, under cold stress. Interestingly, after being exposed to cold, the JA-Ile contents in the scions of Hetero and particularly Hohab were clearly higher than those in the Holyc scions (Fig. 3c). The JA/JA-Ile content, however, increased dramatically in the Hetero and Hohab roots while remaining practically unchanged in the Holyc root after exposure to cold stress. There was a 7.16- and 13.53-fold increase in JA-Ile in Hetero and Hohab rootstock (Fig. 3d). This suggested that JA in *S. habrochaites* rootstock is likely to transport to the scion to enhance JA response.

### Exogenous jasmonic acid application enhances cold tolerance of grafted tomato seedlings

**Figure 4:**
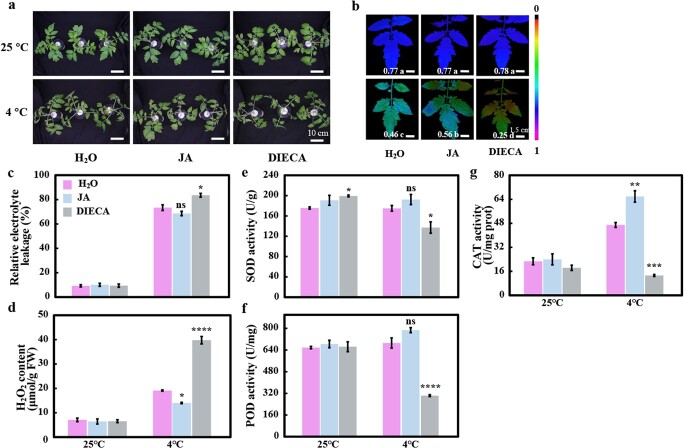
Effects of exogenous JA and DIECA on the cold tolerance of Holyc. **a** Representative image of JA- and DIECA-treated tomato grafts after cold stress. **b***F*_v_*/F*_m_. Means denoted with different letters differ significantly at *P* < .05. **c** Relative electrolyte leakage. **d** H_2_O_2_ content. **e**–**g** SOD (**e**), POD (**f**), and CAT (**g**) activities. At the five-leaf stage, *S. lycopersicum* homografts (Holyc) were treated with deionized water, 200 μM JA, or 200 μM DIECA for 12 hours before they were subjected to cold stress at 4°C. Stress indicators were assessed 48 hours after cold treatment. Data are means of three biological replicates (± standard deviations) with three technical replicates each. Statistical significance levels in **c**–**g** (Student’s *t*-test): ns, not significant; ^*^P ≤ .05; ^**^P ≤ .01; ^***^P ≤ .001; ^****^P ≤ .0001.

To assess the role of JA in rootstock-induced cold tolerance, we tested whether the cold tolerance of Holyc is enhanced by exogenous JA application. As diethyldithiocarbamate acid (DIECA) inhibits JA biosynthesis, we also examined the effect of DIECA on the cold tolerance of Holyc. In cold-treated grafted seedlings, visible symptoms of cold-induced damage, such as leaf drooping and wilting, were seen. However, pretreatment with 200 μM JA reduced cold-induced symptoms (Fig. 4a). Compared with 48 hours of cold treatment with deionized water, pretreatment with DIECA attenuated the cold tolerance of the grafted seedlings, as seen by a drop in *F*_v_*/F*_m_ and RWC and an increase in REL (Fig. 4b and c, Supplementary Data Fig. S4a). The application of JA increased *F*_v_*/F*_m_ by 22%, while DIECA decreased *F*_v_*/F*_m_ by 45% under 48 hours of cold treatment (Fig. 4b). In addition, exogenous JA decreased REL by 33% and DIECA increased REL by 27% compared with the control plants under cold stress (Fig. 4c). Exogenous JA application significantly reduced H_2_O_2_ and MDA; however, DIECA treatment increased the level of H_2_O_2_ and MDA in grafted tomato seedlings under cold stress (Fig. 4d, Supplementary Data Fig. S4b). Consistently, the activity of antioxidant defense enzymes increased with JA treatment and decreased with DIECA treatment (Fig. 4e–g).

### Genetically blocking jasmonic acid biosynthesis in scion decreases cold tolerance

**Figure 5:**
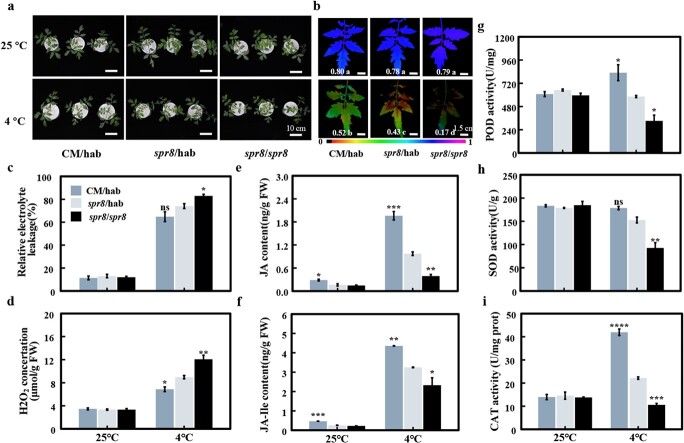
Cold tolerance is impaired in *spr8* scion. **a** Representative images of CM/hab, *spr8*/hab, *spr8*/*spr8* and after cold stress. **b***F*_v_*/F*_m_. Means denoted with different letters differ significantly at *P* < .05. **c** Relative electrolyte leakage. **d** H_2_O_2_ content. **e** JA content in scion. **f** JA-Ile content in scion. **g**–**i** SOD POD(**g**), SOD(**h**), and CAT (**i**) activities. At the five-leaf stage, grafted tomato plants were subjected to cold stress at 4°C for 48 hours before physiological assessment. Data are means of three biological replicates (± standard deviations) with three technical replicates each. (**b**). The *spr8* mutant, with the genetic background of tomato cultivar ‘Castlemart’ (CM), is defective in JA synthesis due to a point mutation in the *LoxD* gene. *S. habrochaites* LA1777 is represented as hab. Seedlings of *spr8* and CM grafted onto hab rootstock and of *spr8* homografted (*spr8*/*spr8*) were treated with cold at 4°C for 48 hours. Statistical significance levels (Student’s *t*-test) in **c**–**i**: ns, not significant; ^*^P ≤ .05; ^**^P ≤ .01; ^***^P ≤ .001; ^****^P ≤ .0001.

Since *LoxD* is a key gene in JA biosynthesis, and it is strongly induced by cold treatment, we used a *LoxD* genetic mutant to determine whether JA is engaged with rootstock-induced cold tolerance. Three graft combinations were generated: tomato *spr8* mutant and its background cultivar (CM) as scion grafted onto the rootstocks of the wild accession LA1777 (CM/hab and *spr8*/hab), and homologous grafted *spr8* mutant (*spr8*/*spr8*). Among them, *spr*8/hab was used as control. Under normal conditions, no obvious difference was observed between *spr8*/*spr8*, *spr8*/hab and CM/hab. However, after cold treatments, *spr8*/*spr8* showed obvious cold damage compared with *spr8*/hab, while CM/hab showed reduced damage, which can be seen by the naked eye (Fig. 5a). Further, measurement also showed that, compared with *spr8*/hab, the *F*_v_*/F*_m_ and RWC of CM/hab were increased, with a decrease in REL in CM/hab scions, and *spr8*/*spr8* was the complete opposite (Fig. 5b–d, Supplementary Data Fig. S5a). Under control and cold stress conditions, the JA and JA-Ile contents in the scion of *spr8*/hab were markedly lower than in those of CM/hab. However, it is worth noting that more JA/JA-Ile was also detected in spr8/hab scion than in spr8/spr8 scion, which could be due to the upward transportation of JA in LA1777 rootstock mentioned above (Fig. 5e and f). In addition, physiological responses to cold stress were compared between *spr8*/*spr8*, *spr8*/hab, and CM/hab. After 48 hours of 4°C treatment, REL and MDA contents in the scions of CM/hab were significantly lower than those in *spr8*/hab (Fig. 5c, Supplementary Data Fig. S5b). We also determined the activity of the antioxidant defense enzymes and it was found that the difference in activity was consistent with the difference in JA/JA-Ile content in scion, indicating that CM/hab was stronger than spr8/hab, and further stronger than spr8/spr8. (Fig. 5g–i). The findings suggest that JA plays a key role in *S. habrochaite* rootstock-induced cold tolerance.

## Discussion

In tomato cultivation, *S. habrochaites*-derived rootstocks are frequently used to improve the scion performance of commercial cultivars, including cold tolerance [30, 31]; however, the underlying mechanism remains unclear. Here, we demonstrated that *S. habrochaites* rootstock confers tomato cold tolerance by enhancing JA accumulation in scions via root-to-shoot communication.

### *Solanum habrochaites*-derived rootstocks improve tomato cold tolerance

Grafting over hardy rootstocks is an effective strategy for strengthening a plant’s resistance to climatic conditions [6, 32]. LA1777 as a representative cold-tolerant germplasm from *S. habrochaites* might be utilized as rootstock to enhance the cold tolerance of tomato grafts. Previous work using LA1777 as rootstock to improve tomato cold tolerance mostly focused on suboptimal temperature (15°C) treatment of whole seedlings or roots [2, 31]. Our data on several stress-related indicators clearly showed that LA1777 rootstock improves the performance of cultivated tomato scions under cold stress (4°C). Tomato seedlings with LA1777 rootstock exhibited less wilting, a lower *F*_v_*/F*_m_ reduction, and a lower REL increment when exposed to 4°C (Fig. 1). Cold stress results in dehydration and wilting, which can be reflected by RWC. Hetero seedlings showed higher RWC compared with Holyc seedlings (Fig. 1e). Plants produce a large amount of ROS under cold stress, which causes oxidative damage to the cell membrane [33]. Antioxidant enzymes, such as POD, SOD, and CAT, function as ROS scavengers, lowering their concentrations and alleviating oxidative damage induced by low temperature. Increasing evidence suggests that grafting can confer cold resistance in plants by regulating the activities and expression profiles of the antioxidative system [34, 35]. Hetero seedlings showed higher activities of POD, SOD, and CAT, and lower ROS and MDA contents compared with Holyc seedlings (Fig. 1f–j).

*Solanum habrochaites* LA1777 rootstock improves the growth and physiological performance of tomato under suboptimal temperature stress [2]. Therefore, it would not be surprising that LA1777 rootstock can also improve tomato plant performance under more severe conditions in our study (4°C). But why does this happen? Our further analysis indicated that JA appears to be crucial to this process.

### Jasmonic acid is involved in *Solanum habrochaite* rootstock-induced cold tolerance

Our findings revealed that activated JA accumulation and robust JA signaling are critical for scion tolerance with *S. habrochaite* rootstock. A variety of phytohormones can be involved in regulating the expression of low-temperature-responsive genes in plants [20]. Jasmonate, the major activator of a subset of cold-regulated genes, has been found to function in modulating tomato plant responses to cold stress [18]. The RNA-seq analysis of homo- and heterograft scions revealed that the JA biosynthesis and signaling pathways were enhanced when *S. habrochaites* LA1777 was used as rootstock, especially after cold exposure, which was further verified by RT–qPCR (Fig. 2), to confirm the correlation between JA and grafted plants. Exogenous hormones or their synthesis inhibitors have been widely used to verify the function of hormones [36]. Wang *et al*. [37] found that exogenous JA application alleviates the detrimental consequences of cold stress, most likely through upregulating the CBF pathway. In our study, similarly, exogenous JA was found to be able to reduce the cold sensitivity of cultivated tomato; in contrast, exogenous application of the JA inhibitor DIECA attenuated the stress tolerance of tomato (Fig. 4, Supplementary Data Fig. S4). Additionally, we used one transgenic line (*spr8*), in which there is a defect in 13-lipoxygenase of the JA production pathway, in an effort to better understand the function of JA in tomato cold tolerance [21]. Lower levels of JA/JA-Ile in mutant scions were discovered to decrease their tolerance to cold stress when *spr8* transgenic plants were compared with their wild-type counterparts (Fig. 5). It is important to note that, under cold stress, *spr8*/hab scions also accumulated a certain amount of JA/JA-Ile compared with *spr8/spr8* scions, so we had reason to suspect that JA in the rootstock was transported upward to the scion. All these results suggested the involvement and importance of JA in *S. habrochaites* LA1777 rootstock-mediated cold tolerance of tomato scion.

Furthermore, our study reveals that the MYC2-dependent JA signaling pathway is critical for regulating the antioxidant defense system in grafted tomato plants under cold stress, adding another piece of evidence supporting *S. habrochaites* LA1777 rootstock-induced cold tolerance in tomato grafts. MYC2 is the main regulator of the JA signaling pathway, and can bind to the promoter of JA-responsive genes directly [38]. Several investigations have demonstrated that JA inhibits cold-induced ROS generation in plants by boosting the manufacture of antioxidative compounds, such as glycine betaine and polyamines. Upregulation of *ARG1*, *ARG2*, *ADC*, and *ODC* by *MYC2* is involved in MeJA-induced polyamine biosynthesis, thus enhancing chilling tolerance in tomato fruit [39]. MYC2-SlGSTU24 seems to be a key module that keeps ROS homeostasis in tomato under cold conditions [40]. Nevertheless, our understanding of JA-enhanced plant cold tolerance remains limited. To this end, we investigated the shared genes between our CRGs and the 665 MYC2 targets previously revealed by ChIP experiments [29]. Among them, there are three genes encoding antioxidant enzymes upregulated in Hetero but not in Holyc. JA-triggered expression of *SOD*, *POD*, and *CAT* would ultimately enable plants to eliminate cold-induced ROS more efficiently. The results from the comparison of *spr8* and its background cultivar (CM) also supported the idea that *SOD*, *POD*, and *CAT* could be direct targets of MYC2. Compared with its wild-type counterpart, the *spr8* mutant produced a lower level of JA/JA-Ile and showed lower activities of *SOD*, *POD*, and *CAT* under cold treatment (Fig. 5). These results indicate that JA could elevate antioxidant enzyme activity and enhance ROS scavenging potential.

### Mobile signals in jasmonic acid-mediated cold tolerance of tomato grafts

There could be different types of mobile signals by which the rootstock and scion can communicate in the cold response. The involvement of jasmonate in the cold stress mechanism is of special interest, especially as a long-distance signal. According to our research, *S. habrochaites* LA1777 rootstock-sourced JA itself may function as a long-distance signal that causes JA accumulation and cold tolerance in *S. lycopersicum* LA4024 leaves. The basal JA content is higher in LA1777 than in LA4024, which may be attributed to the unique traits of *S. habrochaites*, such as hairy shoots and strong herbivorous insect resistance [41]. Moreover, we evaluated hormone levels of roots and shoot tissues of grafted plants (Fig. 3). Generally, grafting often had little noticeable impact on JA concentrations in tissues and transport pathways, because the JA/JA-Ile content in Holyc scion is not significantly different from that in Hetero under normal conditions. It is worth noting that the increase in JA/JA-Ile content in Hetero scions co-occurred with an increase in JA/JA-Ile in Hetero rootstocks, but the content of JA in Hetero and Hohab rootstocks was significantly different. Meanwhile, the increase in JA/ JA-Ile in Holyc scions was much lower than that in Hetero scions, and the JA/JA-Ile content in Holyc rootstocks remained unchanged. As a result, it is possible that some distant signals originating from the rootstock can be involved in inducing JA synthesis in scions under cold stress. We suspected that JA itself or other mobile signals in the rootstock can activate JA accumulation in the scion through long-distance transportation. After all, it has been reported that rice can resist salt and drought stress by synthesizing JA in roots and transporting it to scions [42, 43]. Likewise, De Ollas *et al*. [44] demonstrated that root-derived JA is implicated in the shoot response of grafted tomato under drought stress. These findings imply that, in response to cold stress, *S. habrochaites* rootstock-derived JA may operate as a long-distance signal, inducing JA accumulation and cold tolerance in grafted tomato.

Although JA may be the first suspect, other signals cannot be excluded. In pumpkin, melatonin can induce MeJA and H_2_O_2_ synthesis in watermelon scion to improve plant cold tolerance [5]. Nevertheless, our results showed that Hetero scions accumulated less H_2_O_2_ than Holyc scions after cold treatment, which seems to be opposite to the results in cucurbitaceous species. However, it should not be ignored that H_2_O_2_ has dual functions as a signal molecule and a toxic metabolite [45]. Therefore, melatonin may also act as a signal molecule in the early stage of cold response. Other mobile signals that should be considered are mRNAs or small RNAs, small peptides or even proteins, and other phytohormones or metabolites [46].

Fortunately, the availability of an introgression population of *S. habrochaites* LA1777 [47] would enable us to identify the key natural variations underlying rootstock-mediated scion cold tolerance. Recently, Guo *et al*. [48] found that nucleotide polymorphism in *cis*-regulatory regions is crucial for different cold sensitivity between wild and cultivated tomato plants. Our previous study also narrowed down the candidate introgression lines [4], which can serve as a good starting point for the genetic dissection of *S. habrochaites* LA1777-mediated scion tolerance in tomato.

### Conclusions

**Figure 6:**
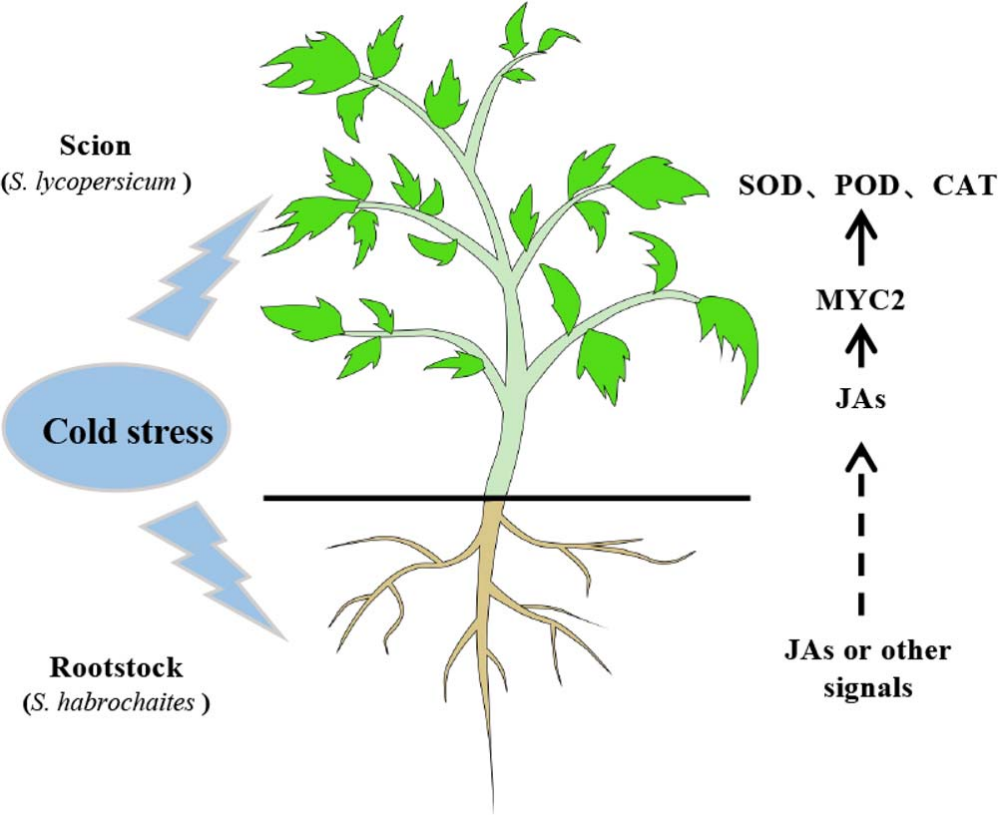
A proposed model of *S. habrochaites* LA1777 rootstock-mediated improvement of cold tolerance of cultivated tomato scion. When grafts are exposed to cold stress, JA accumulates in the scion due to a long-distance JA signal or other signals from the rootstock, and this stimulates JA biosynthesis genes in the scion, such as *MYC2*. *MYC2* then activates the expression of antioxidant enzyme genes (*SOD*, *POD*, *CAT*), leading to enhanced ROS scavenging and improved cold tolerance.

The processes behind rootstock-induced scion tolerance to cold stress are largely unknown to date. We demonstrated that *S. habrochaites* LA1777 rootstock promotes JA accumulation in the scion, resulting in elevated antioxidant enzyme activity and enhanced ROS scavenging potential, which is likely due to JA activation of MYC2 targets such as *SOD*, *POD*, and *CAT*. We proposed that JA or other mobile signals from *S. habrochaites* rootstock can activate the JA signaling pathway and subsequently improve scion cold tolerance by altering the antioxidative defense mechanism (Fig. 6).

## Materials and methods

### Plant materials

In this research, four different tomato accessions were utilized, including the wild accession LA1777 (*S. habrochaites*) and three cultivated genotypes [LA4024, *spr8* mutant and its genetic background cultivar ‘Castlemart’ (CM)]. The mutant *spr8* is a dominant negative mutant in the CM background, which has a point mutation in *LoxD* that encodes the 13-lipoxygenase of the JA synthesis pathway [21].

Germinated tomato seeds were sown in 50-well sowing trays filled with peat:vermiculite:perlite (3:1:1). Germinated seeds of *spr8*, LA4024, and CM were sown 7 days (*spr8*) or 10 days (LA4024 and CM) later than LA1777 to ensure similar stem size of seedlings during grafting. The seedlings were raised in growth chambers with a temperature of 25/18°C (day/night), a photoperiod of 14 hours light/10 hours dark, and photosynthetic photon flux density (PPFD) of 400 μmol m^−2^ s^−1^. Splice grafting was performed when the scion (LA4024, CM, or *spr8*) was at the two-leaf stage, where the scion was attached at 1–2 cm above the ground of the rootstock, LA1777 (hab). Ten days after grafting, the roots were gently washed to remove soil and grown hydroponically in half-strength Hoagland solution and the solution was renewed every 5 days.

### Experimental design

To evaluate the impacts of LA1777 rootstock on scion tolerance to cold stress, Holyc and Hetero plants with five true leaves were transplanted into growth chambers kept at 23°C (control) or 4°C (cold treatment). *F*_v_*/F*_m_ and REL were measured at 0, 3, 6, 48, and 120 hours after cold treatment. Samples of roots or leaves were harvested to determine biochemical indicators.

To determine the impacts of exogenous JA on cold resistance of Holyc seedlings, plants were pretreated with distilled water (control), JA (Sigma–Aldrich) at 200 μM, and DIECA, a JA biosynthesis inhibitor, at 200 μM, on leaves. JA and DIECA were diluted with distilled water at a ratio of 1/10 000 (v/v) after being dissolved in ethanol and Tween 20, respectively. Each plant with five true leaves was sprayed with 20 ml of the chemical solution or distilled water (control). After pretreatment for 12 hours, the plants underwent cold treatment at 4°C. *F*_v_*/F*_m_ and REL were evaluated after 48 hours of cold treatment, and the roots or leaves were taken for biochemical indicator measurement.

To investigate the role of endogenous JA in LA1777 rootstock-mediated cold tolerance of tomato scions, the *spr8* mutant and its wild-type (CM) were grafted onto LA1777 rootstock (*spr8*/hab and CM/hab), respectively, and another homologous grafted *spr8* mutant (*spr8*/*spr8*), *spr8*/hab was used as control. After healing, grafted plants with five true leaves were subjected to cold treatment at 4°C. Similarly, *F*_v_*/F*_m_, REL and related biochemical parameters were measured 48 hours after cold treatment. Meanwhile, roots and fully expanded young leaves were immediately collected, frozen in liquid nitrogen, and stored at −80°C for hormone determination.

### Chlorophyll fluorescence (*F*_v_*/F*_m_) measurement

*Fv/Fm* was monitored by imaging PAM (IMAG-MAX/L, Germany). With three to five readings per replication, measurements were taken in the middle of the second completely developed leaf from the bottom. Thirty minutes of dark treatment was provided before observing the readings [49].

### Leaf relative water content and relative electrical conductivity assays

With leaf disks 5 mm in diameter and weighing roughly 500 mg in FW from the third fully expanded leaf, RWC was calculated. The disks were weighed immediately to get their FW, and then they were put in Petri dishes with deionized water for 24 hours to get their turgid weight (TW). The disks were then dried for 24 hours at 80°C to determine their dry weight (DW). The formula for RWC was (FW − DW)/(TW − DW) × 100.

REL was measured following a previous study [50]. In brief, 0.1 g of leaf sample was divided into pieces measuring 1 cm^2^ and then shaken for 4 hours at 22°C after being rinsed with deionized water. The conductivity of the incubation solution was measured as EC1. Leaf samples were then boiled for 30 minutes and the conductivity of the solution was measured as EC2. The REL was calculated as the ratio EC1/EC2.

### Determination of hydrogen peroxide and malondialdehyde contents

H_2_O_2_ was measured using a kit from the Nanjing Jiancheng Institute of Biological Engineering, China. However, MDA was estimated with the method of Amin *et al*. [51]. With 2 ml of extraction solution (1/15 M phosphate buffer, pH 7.8, 0.2 mM EDTA, and 2% polyvinylpyrrolidone), leaf tissues weighing 0.1 g were extracted. The supernatant from the centrifugation of the samples at 12 000 g for 20 minutes was used to measure MDA. A combination containing 0.65% (w/v) thiobarbituric acid (TBA) and 10% trichloroacetic acid (TCA) was combined with 0.5 ml of supernatant extract and then heated in a water bath at 100°C for 15 minutes to stop the process. After centrifuging the reaction mixture at 10 000 g for 10 minutes, the absorbance at 532, 600 and 450 nm was recorded. MDA content was calculated as 6.45(OD_532_ − OD_600_) − 0.56OD_450_.

### Transcriptome and RT–qPCR analysis

RNA-seq analysis was performed using root and second fully expanded leaf samples from the five-leaf stage of Holyc, Hetero, and Hohab without (CK) or with 3 hours of 4°C cold treatment (LT). The RNA-seq samples were named HolycCK, HeteroCK, HohabCK, HolycLT, HeteroLT, and HohabLT, respectively. A total of 0.5 g leaf or root sample was ground into powder in liquid nitrogen. Total RNA was extracted using Trizol reagent (Invitrogen, Carlsbad, USA). Qualified RNA samples were sent for transcriptome sequencing (Megi Bio, Shanghai, China). The Sol Genomic Network database (https://solgenomics.net) was used for gene expression analysis. RPKM (reads per kilobase of transcript per million reads mapped) was used to calculate gene expression. DESeq2 software was used to determine differentially expressed genes (DEGs), using the criteria of log_2_ (fold-change value) >1, and *P*-value <.05. GO enrichment and the KEGG pathway analysis of DEGs was performed using Cluster Profile software [52].

Quantitative PCR analysis (RT–qPCR) used reverse transcription to create cDNA from the whole RNA. For each sample, three biological replicas were employed. RT–qPCR was performed with the QuantStudio 7 Flex system (Applied Biosystems, USA) using TransScript^®^ Green Two-Step qRT-PCR SuperMix (Transgen Biotech Inc., Beijing, China). The tomato actin gene was used as internal control, and the relative expression level was calculated as previously described [53]. Primers used in RT–qPCR are listed in Supplementary Data Table S1. Data are the means of three biological replicates with four technical replicates each.

### Determination of antioxidative enzyme activities

A fresh leaf sample weighing 0.1 g was homogenized in 2 ml of phosphate buffer (pH 7.6) supplemented with 1 mM EDTA and 4% (w/v) polyvinylpyrrolidone before being incubated at 4°C for 10 minutes to test antioxidant enzyme activity. After centrifuging the homogenate (12 000 g) at 4°C for 15 minutes, the supernatant was utilized to estimate the enzyme concentration. SOD activity was measured according to Kumar *et al*. [54]: an aliquot of 20 μl of the enzyme extract was combined with 3 ml of the SOD reaction mixture, which contained 0.25 ml of distilled water and 1.5 ml of 50 mM phosphate buffer, pH 7.8, 0.75 mM nitro blue tetrazolium (NBT), 130 mM methionine, 0.02 mM riboflavin, and 0.3 ml of 0.1 mM EDTA-Na. Absorbance was measured at 560 nm. POD was measured according to Chakraborty *et al*. [55]. A 20-μl aliquot of the enzyme extract was mixed with 3 ml of POD reaction mixture [2.9 ml 0.05 mM phosphate buffer, pH 5.5, 1 ml 0.05 M guaiacol, and 1 ml 2% (w/v) H_2_O_2_]. Absorbance was recorded at 470 nm. The CAT activity was assessed using the catalase test kit from the Nanjing Jiancheng Institute of Biological Engineering.

### Determination of jasmonic acid and jasmonic acid-isoleucine content

In accordance with a methodology that has been published [56], leaf and root samples were crushed into a powder in liquid nitrogen and sent for measurement of JA and JA-Ile by liquid chromatography-mass spectrometry (ESI–HPLC–MS) (Wuhan Triploid Biotechnology Co. Ltd., China). A 0.2-g sample was extracted with 10× acetonitrile (v/v) overnight at 4°C. The extracts were centrifuged at 12 000 g for 5 minutes. Following centrifugation, the pellet was extracted using an additional 5× acetonitrile (v/v). The supernatants from the two extractions were pooled, along with 15 mg of C18 filling. After being shaken vigorously for 30 seconds, the solution was centrifuged at 10 000 g for 5 minutes. The supernatant was collected and evaporated under a nitrogen flow. The dried material was dissolved in 200 μl of methanol, put through a filter with 0.22-m pore size, and then put in a freezer at −20°C.

### Statistical analysis

Statistical analysis was performed and plots were generated using Rstudio 4.03 [57]. Data from three biological replicates were used to calculate the means and standard deviations. The data were subjected to Duncan’s multiple range test and Student’s *t*-test or one-way ANOVA, and *P* <.05 was considered to indicate a significant difference. Final processing of figures was performed with Adobe Photoshop 6.0 software (Adobe Systems, San Jose, CA).

## Acknowledgements

We thank Prof. Chuanyou Li (Institute of Genetics and Developmental Biology, Chinese Academy of Sciences, Beijing, China) for providing the *spr8* mutant and its background parent used in this study. This research was supported by the National Key Research and Development Program (2019YFD1001900, 2022YFE0100900) and the Natural Science Foundation of Hubei Province (2019CFA017).

## Author contributions

L.W., B.W., Z.B., and B.O. conceived and designed the experiments. G.C., H.C., Y.P., H.S., G.L., S.G., and D.X. performed the experiments and analyzed the data. L.W., B.O., and Z.B. wrote the paper.

## Data availability

All the data generated in this study are included in this article and its supplementary information. Data from RNA-seq were submitted to the Sequence Read Archive (SRA) at the National Center for Biotechnology Information (NCBI) under accession number PRJNA868139 (https://www.ncbi.nlm.nih.gov/bioproject/PRJNA868139).

## Conflict of interest

The authors claim to have no conflicts of interest.

## Supplementary data

Supplementary data is available at *Horticulture Research* online.

## Supplementary Material

Web_Material_uhac227
