# Supplementary material for: The essential role of jasmonate signaling in Solanum habrochaites rootstock-mediated cold tolerance in tomato grafts
Source: Hortic Res. 2022 Oct 11;10(1):uhac227. doi: 10.1093/hr/uhac227 (PMC9832872; doi:10.1093/hr/uhac227)
Supplement: Web_Material_uhac227 [file web_material_uhac227.zip › Supplementary date.pptx]

## Slide 1
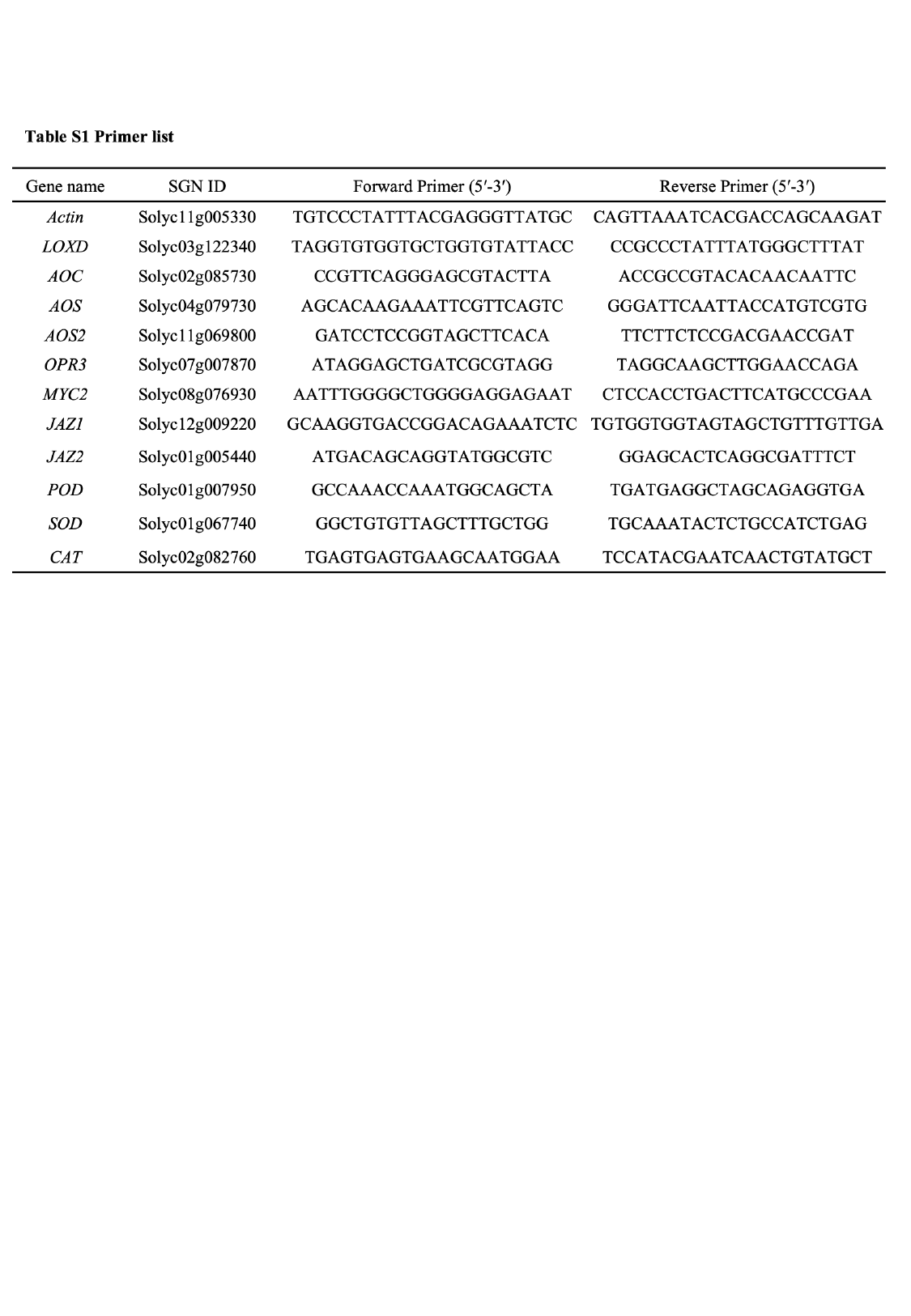

## Slide 2
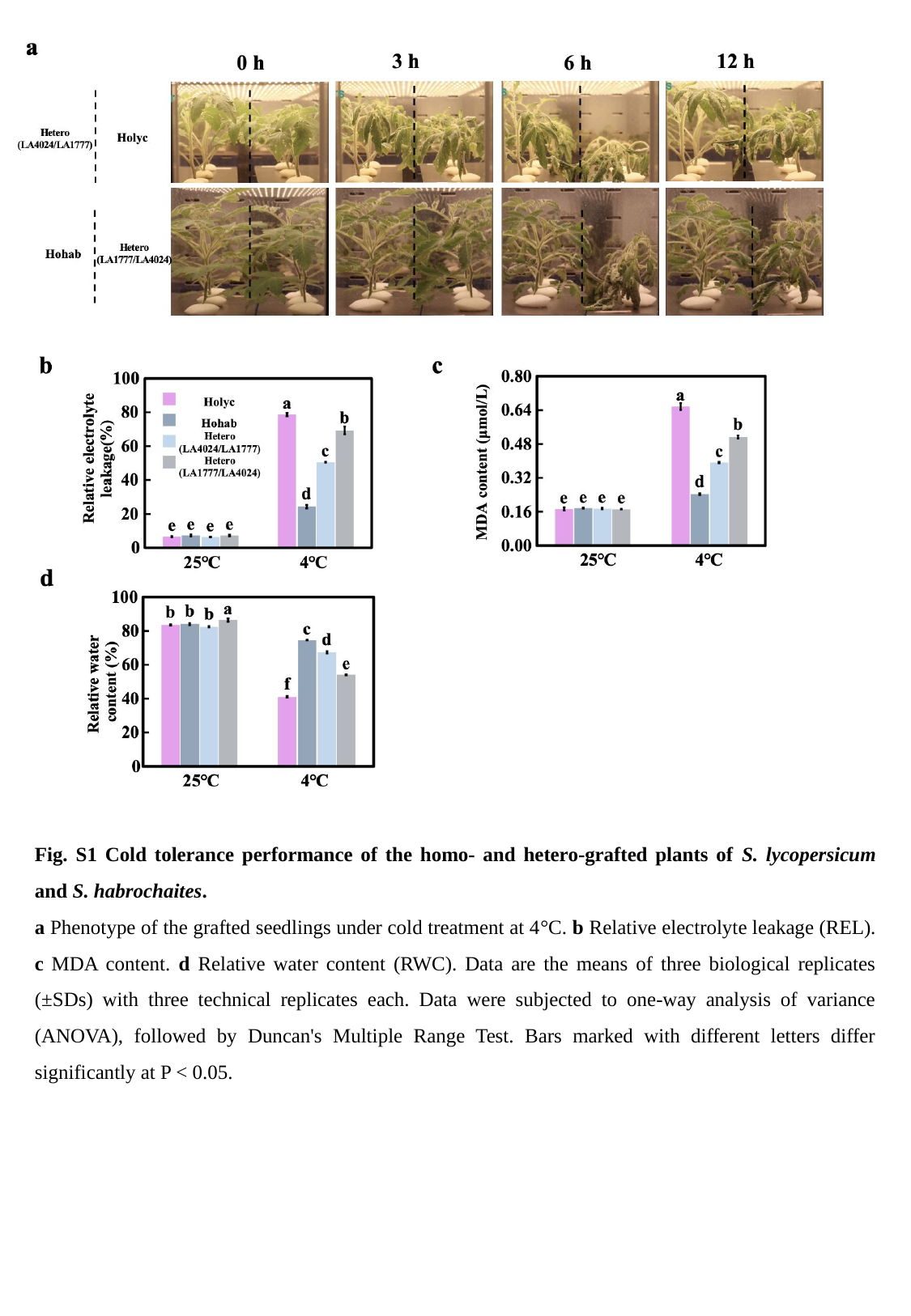

Fig. S1 Cold tolerance performance of the homo- and hetero-grafted plants of S. lycopersicum and S. habrochaites.
a Phenotype of the grafted seedlings under cold treatment at 4°C. b Relative electrolyte leakage (REL). c MDA content. d Relative water content (RWC). Data are the means of three biological replicates (±SDs) with three technical replicates each. Data were subjected to one-way analysis of variance (ANOVA), followed by Duncan's Multiple Range Test. Bars marked with different letters differ significantly at P < 0.05.

## Slide 3
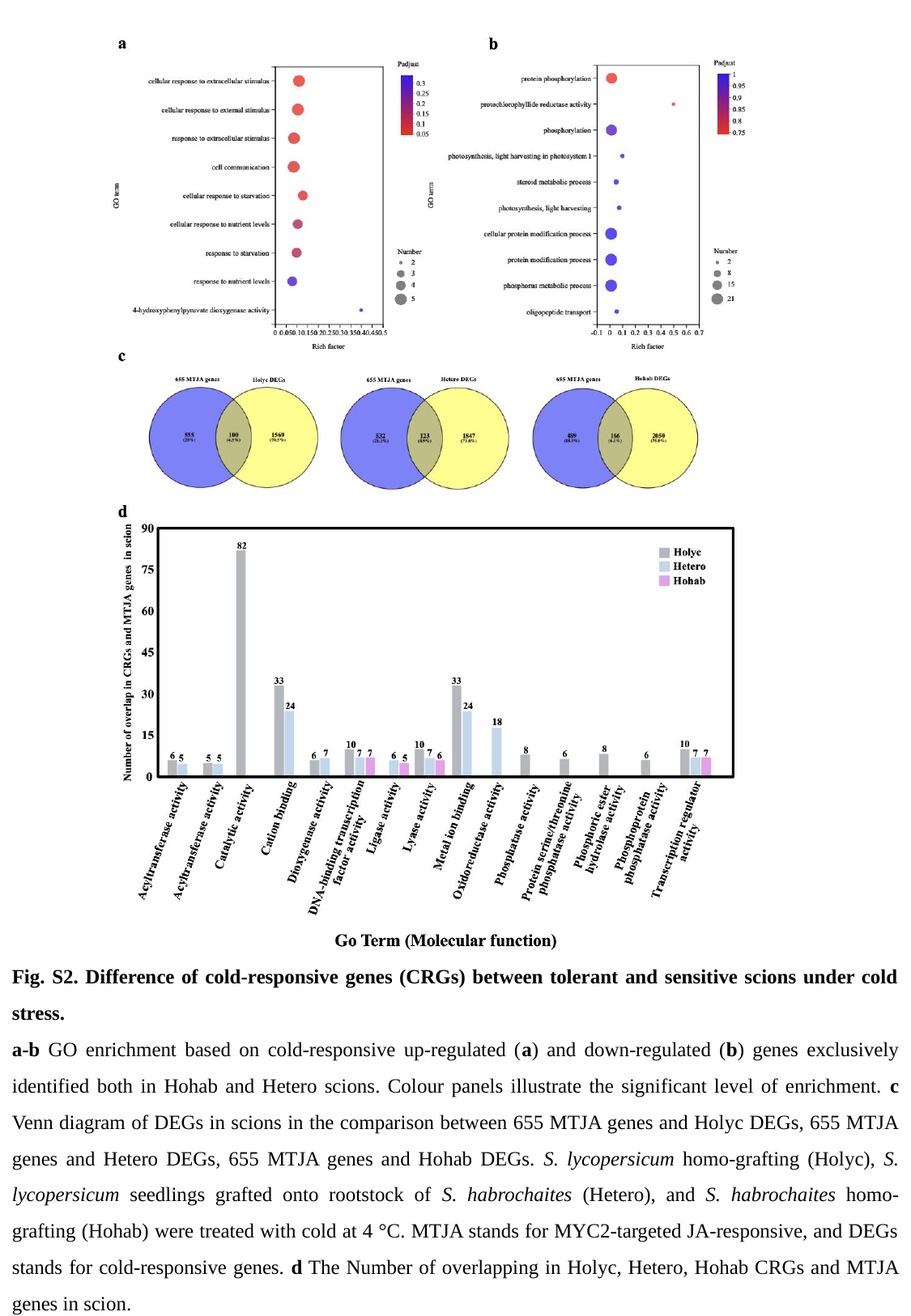

Fig. S2. Difference of cold-responsive genes (CRGs) between tolerant and sensitive scions under cold stress.
a-b GO enrichment based on cold-responsive up-regulated (a) and down-regulated (b) genes exclusively identified both in Hohab and Hetero scions. Colour panels illustrate the significant level of enrichment. c Venn diagram of DEGs in scions in the comparison between 655 MTJA genes and Holyc DEGs, 655 MTJA genes and Hetero DEGs, 655 MTJA genes and Hohab DEGs. S. lycopersicum homo-grafting (Holyc), S. lycopersicum seedlings grafted onto rootstock of S. habrochaites (Hetero), and S. habrochaites homo-grafting (Hohab) were treated with cold at 4 °C. MTJA stands for MYC2-targeted JA-responsive, and DEGs stands for cold-responsive genes. d The Number of overlapping in Holyc, Hetero, Hohab CRGs and MTJA genes in scion.

## Slide 4
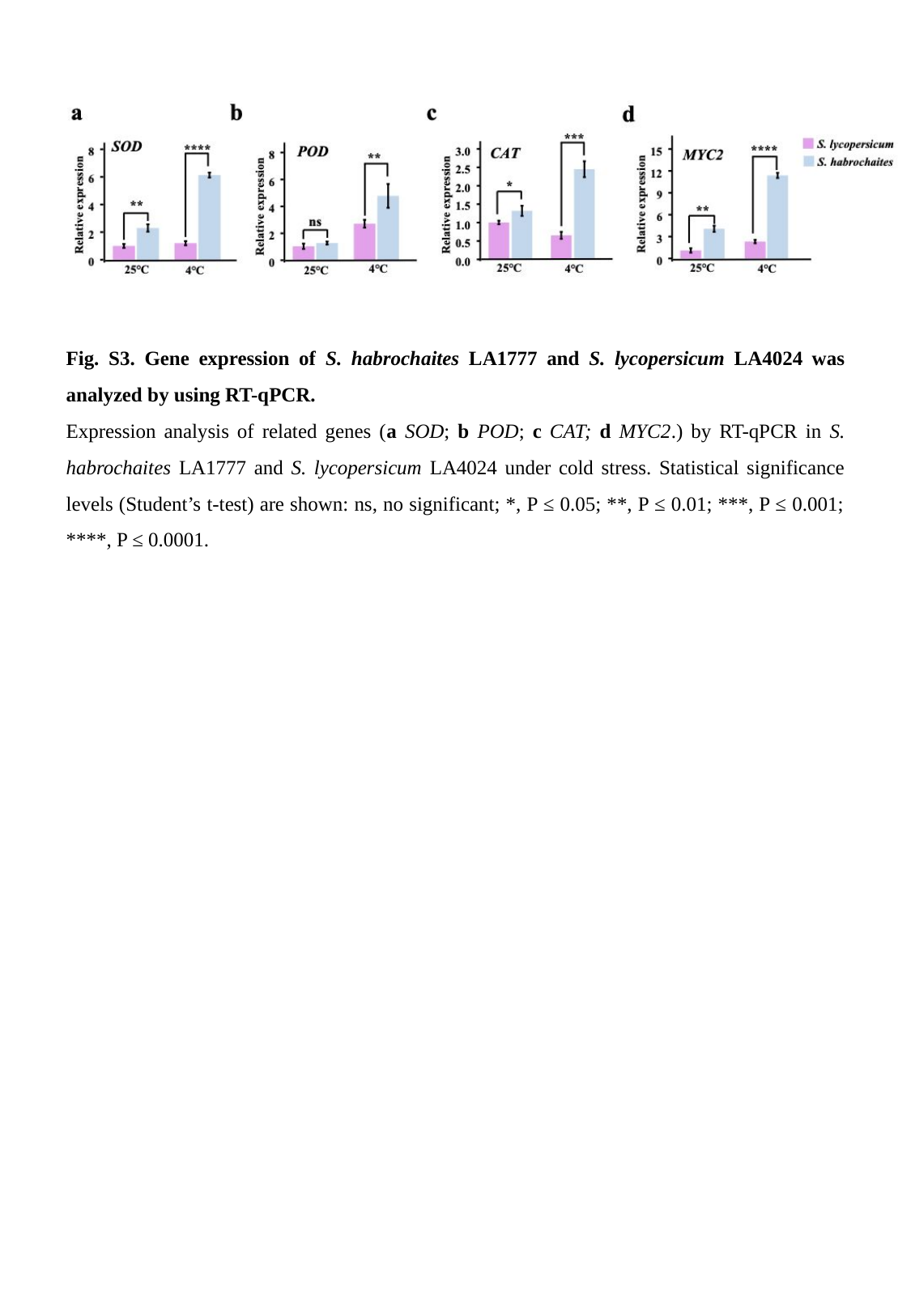

Fig. S3. Gene expression of S. habrochaites LA1777 and S. lycopersicum LA4024 was analyzed by using RT-qPCR.
Expression analysis of related genes (a SOD; b POD; c CAT; d MYC2.) by RT-qPCR in S. habrochaites LA1777 and S. lycopersicum LA4024 under cold stress. Statistical significance levels (Student’s t-test) are shown: ns, no significant; *, P ≤ 0.05; **, P ≤ 0.01; ***, P ≤ 0.001; ****, P ≤ 0.0001.

## Slide 5
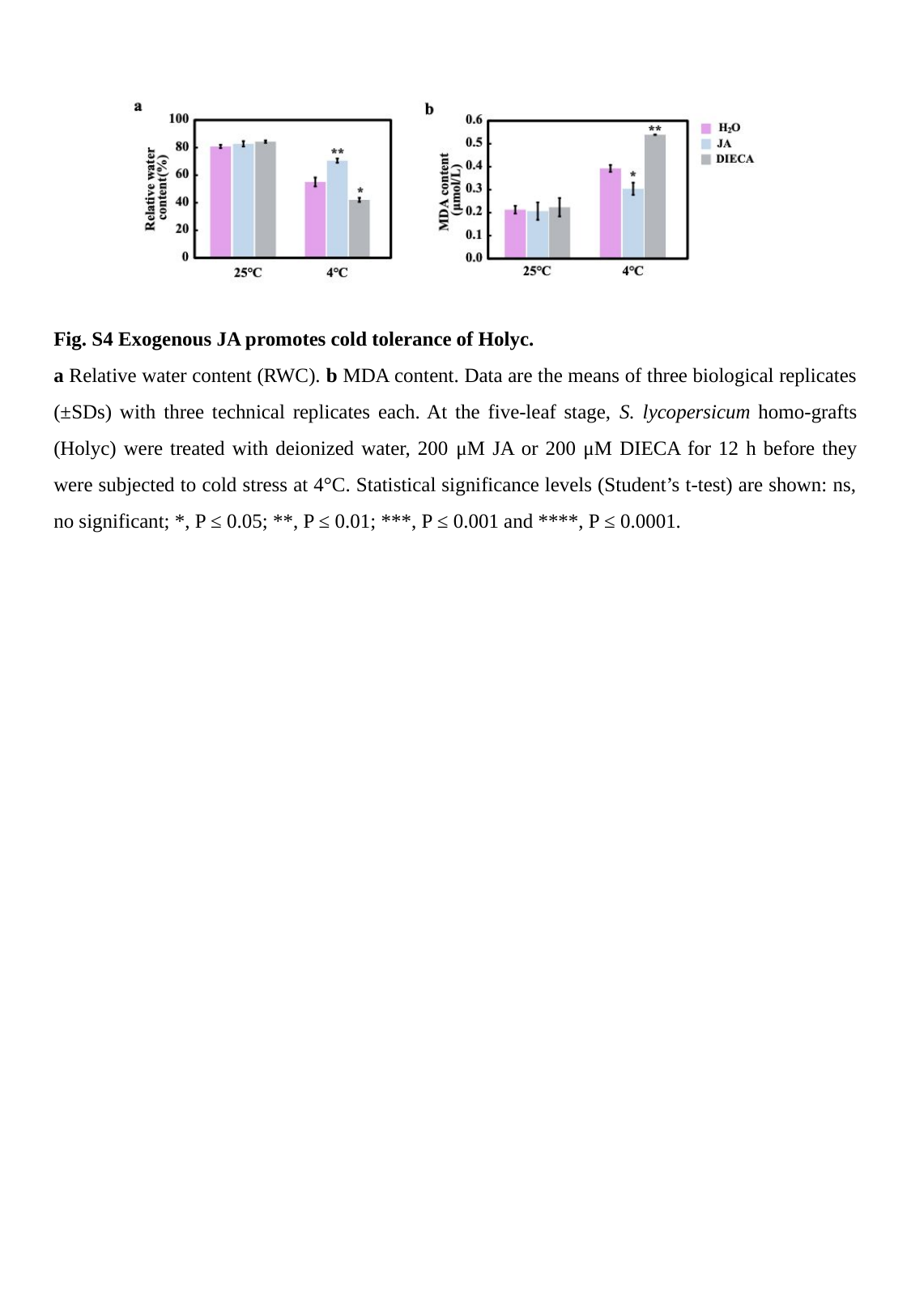

Fig. S4 Exogenous JA promotes cold tolerance of Holyc.
a Relative water content (RWC). b MDA content. Data are the means of three biological replicates (±SDs) with three technical replicates each. At the five-leaf stage, S. lycopersicum homo-grafts (Holyc) were treated with deionized water, 200 μM JA or 200 μM DIECA for 12 h before they were subjected to cold stress at 4°C. Statistical significance levels (Student’s t-test) are shown: ns, no significant; *, P ≤ 0.05; **, P ≤ 0.01; ***, P ≤ 0.001 and ****, P ≤ 0.0001.

## Slide 6
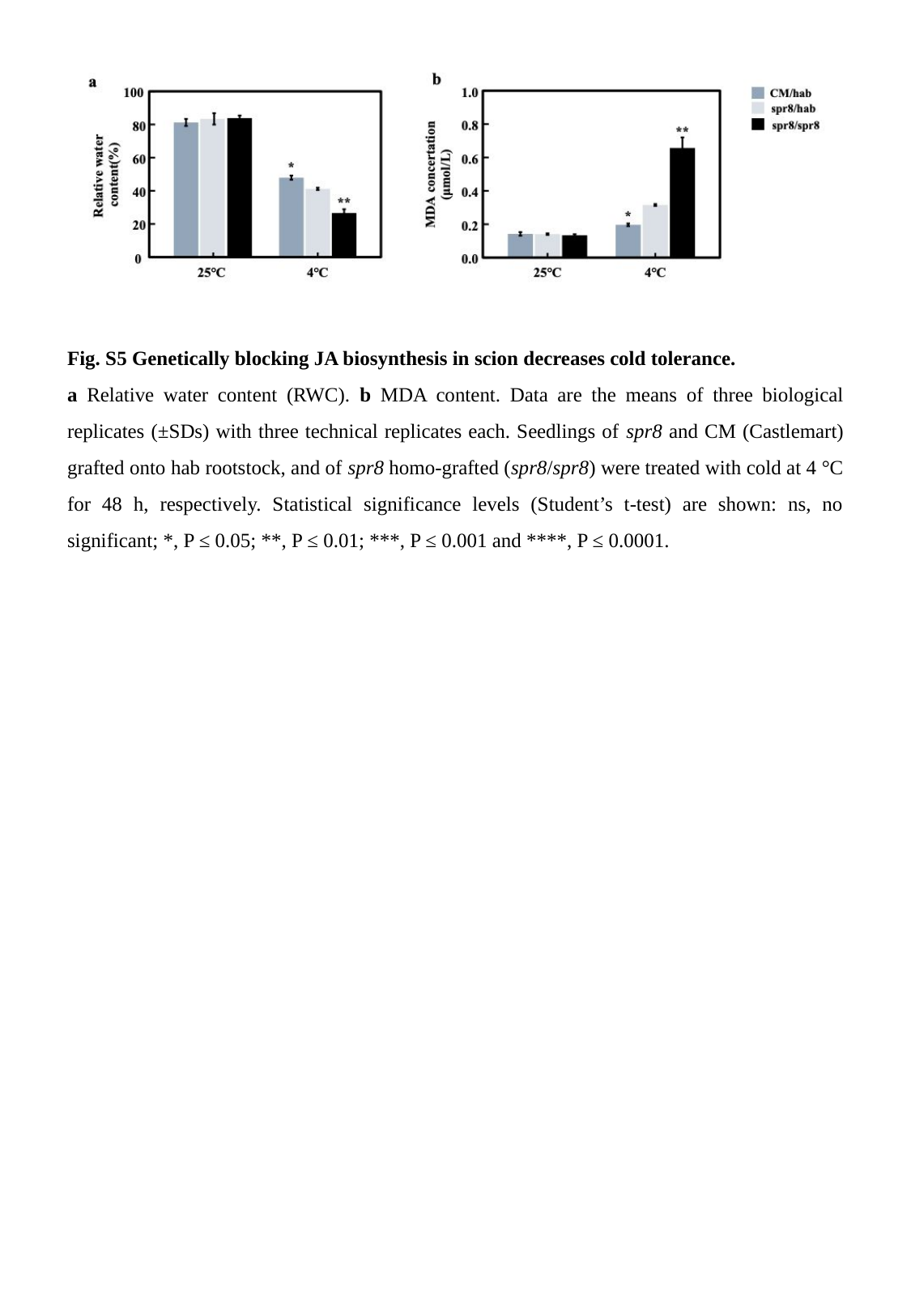

Fig. S5 Genetically blocking JA biosynthesis in scion decreases cold tolerance.
a Relative water content (RWC). b MDA content. Data are the means of three biological replicates (±SDs) with three technical replicates each. Seedlings of spr8 and CM (Castlemart) grafted onto hab rootstock, and of spr8 homo-grafted (spr8/spr8) were treated with cold at 4 °C for 48 h, respectively. Statistical significance levels (Student’s t-test) are shown: ns, no significant; *, P ≤ 0.05; **, P ≤ 0.01; ***, P ≤ 0.001 and ****, P ≤ 0.0001.
